# Research Progress on Precision Tool Alignment Technology in Machining

**DOI:** 10.3390/mi15101202

**Published:** 2024-09-28

**Authors:** Qimeng Liu, Junxiang Jiang, Wencui Xiu, Zhe Ming, Bo Cui, Liang Zheng, Jian Wang, Liyan Qi

**Affiliations:** 1 School of Mechanical and Civil Engineering, Jilin Agricultural Science and Technology University, Jilin 132101, China; 2 Advanced Manufacturing Technology Engineering Research Center for Key Components of Agricultural Machinery Equipment, Jilin Agricultural Science and Technology University, Jilin 132101, China; 3 Agricultural Smart Picking Image Recognition Innovation Team, Jilin Agricultural Science and Technology University, Jilin 132101, China

**Keywords:** tool setting, trial cutting, tool setting method, tool setting device

## Abstract

In the field of numerical control machining, tool alignment technology is a key link to ensure machining accuracy and quality. Tool alignment refers to determining the correct position of the tool relative to the workpiece, and its accuracy directly affects the precision of part machining. With the development of precision machining technology, the research and application of cutting technology are increasingly valued. Tool alignment methods are mainly divided into two categories: contact and non-contact. The contact type tool alignment method relies on direct contact between the tool and the workpiece or tool alignment instrument to measure the position. Among them, the trial cutting method is a traditional contact type tool alignment method that determines the tool position through actual cutting, which is intuitive but inefficient. The contact type tool presetter uses specialized equipment to improve the accuracy and efficiency of tool presetting through contact measurement. The non-contact tool alignment method does not rely on physical contact, while the image method uses image recognition technology to determine the tool position, making it suitable for high-precision applications. The laser diffraction method and the laser direct method use laser technology for non-contact measurement. The laser diffraction method determines the position of the tool by analyzing the diffraction mode of the laser beam, while the laser direct method directly measures the distance between the laser and the tool. This article mainly introduces the classification of tool alignment, commonly used knife alignment methods and common tool alignment devices, as well as the development status of international tool alignment instrument products.

## 1. Introduction

Numerical control machining technology refers to the use of digital programs to control the movement of CNC (numerical control) machine tools, achieving precise machining of workpieces. This technology has become an indispensable part of modern manufacturing due to its high efficiency, high precision, and high degree of automation. Numerical control machining technology is widely used in fields such as aerospace, automotive manufacturing, and mold processing. In the process of CNC machining, tool alignment technology is a key link to ensure machining accuracy. High precision tool alignment can avoid tool collisions and tool damage during the cutting process, and the efficiency of tool alignment can also have a significant impact on the production and processing efficiency of the machine tool [1,2,3,4,5].

Tool alignment, which determines the relative position relationship between the tool and the workpiece, is a fundamental task in CNC machining. The accuracy of the tool directly determines the machining precision, which in turn affects product quality and production costs. Therefore, mastering and applying tool alignment technology is of great significance for improving the quality and efficiency of CNC machining [6,7,8,9,10,11,12,13].

With the continuous advancement of numerical control technology, tool alignment technology is also constantly developing and improving. The development of tool alignment technology has greatly improved the automation level and machining accuracy of CNC machining, from the initial manual tool alignment to semi-automatic tool alignment, and then to modern fully automatic tool alignment systems. The current knife alignment methods are mainly divided into two types according to the operation method: contact knife alignment method and non-contact knife alignment method. This article mainly introduces the classification of knife alignment, commonly used knife alignment methods and common knife alignment devices, as well as the development status of international knife alignment instrument products.

## 2. Contact Type Tool Alignment Method

### 2.1. Trial Cutting Method

The trial cutting tool alignment method is the most widely used manual tool alignment method in machine tool processing applications. When using the trial cutting method for blade measurement, no other auxiliary tools are needed, and only ensuring that the components are tightly installed is sufficient. The advantages are that it is commonly used and easy to operate, with a low cost. The disadvantage is that the production efficiency is relatively low, requiring workers to have high technical skills, leaving cutting marks on the surface of the workpiece, and the accuracy of the tool is low. There are three commonly used methods for trial cutting: (a) Set the tool offset compensation method for tool alignment. (b) Use G54 to set the zero-point alignment method for the workpiece. (c) Use G50/G92 to set the zero-point tool alignment method for the workpiece [14,15,16,17,18,19,20].

### 2.2. Standard Core Shaft, Feeler Gauge, and Block Gauge Tool Method

**Figure 1:**
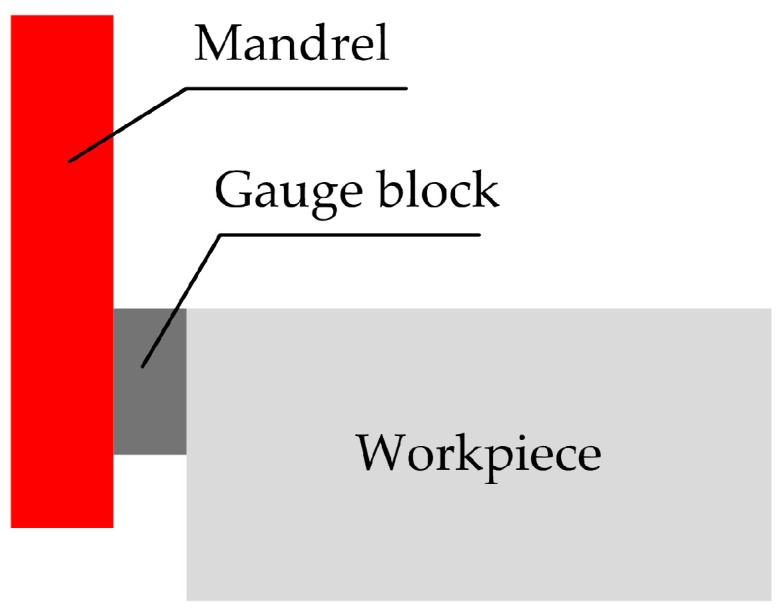
The principle of the standard core shaft trial cutting method.

This method does not require spindle rotation during tool alignment, and only tools such as feeler gauges need to be added between the core shaft and the workpiece to ensure that the feeler gauge cannot move between the core shaft and the workpiece. When calculating the tool position, the thickness of the feeler gauge must be subtracted. The advantages are similar to the trial cutting method, with simple operation and no scratches on the surface of the workpiece. The disadvantages are time-consuming tool setting and low production efficiency [21]. The principle of the standard core shaft trial cutting method is shown in Figure 1.

### 2.3. Use an Edge Finder and a Z-Axis Setter to Adjust the Cutting Method

An edge finder does not cause damage to the surface of the workpiece during the tool alignment process and has the advantages of easy operation and high tool alignment accuracy. It should be used when the workpiece has high straightness [22,23].

The most commonly used edge finders include split rods, digital edge finders, and photoelectric edge finders.

**Figure 2:**
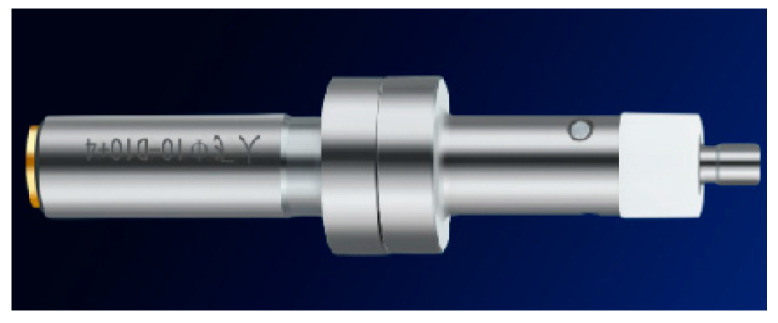
Splitting rod edge finder.

The dividing rod, as shown in Figure 2, requires the spindle speed to be set at around 500 r/min during use, with an accuracy of 0.05 mm, no maintenance required, and a moderate cost.

**Figure 3:**
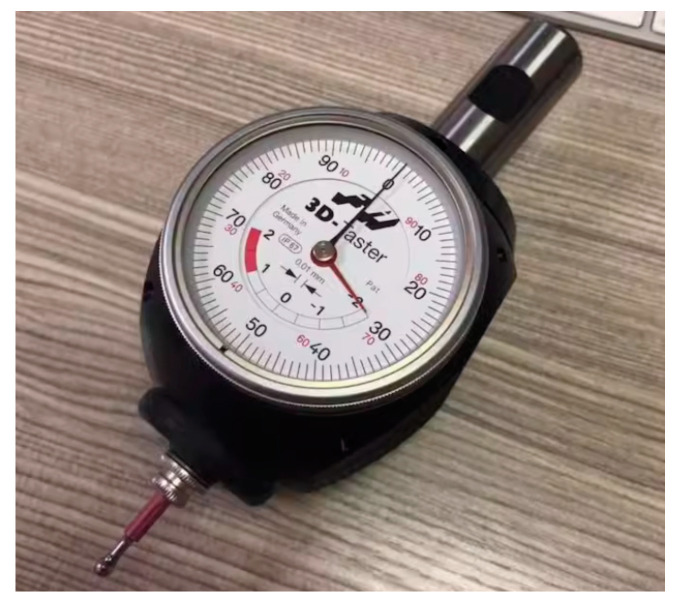
Digital border finder.

The digital edge finder is shown in Figure 3. The advantage is high precision in cutting, with an accuracy of up to 0.01 mm, and simple operation. The disadvantage is that the cost is relatively high.

**Figure 4:**
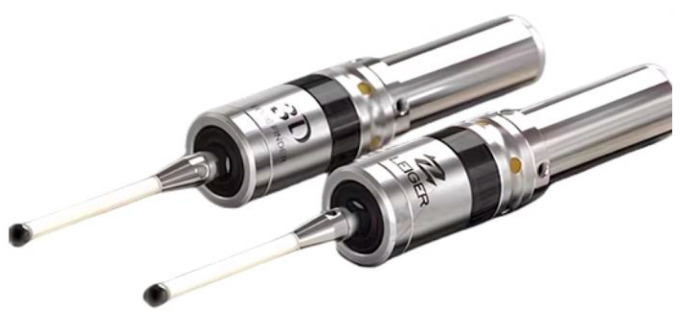
Optoelectronic border finder.

The photoelectric edge finder, as shown in Figure 4, does not rotate the spindle during operation. Advantages: High precision in cutting, high efficiency, and a moderate cost. Disadvantage: The workpiece must have conductivity and cannot be used for non-conductive workpieces.

**Figure 5:**
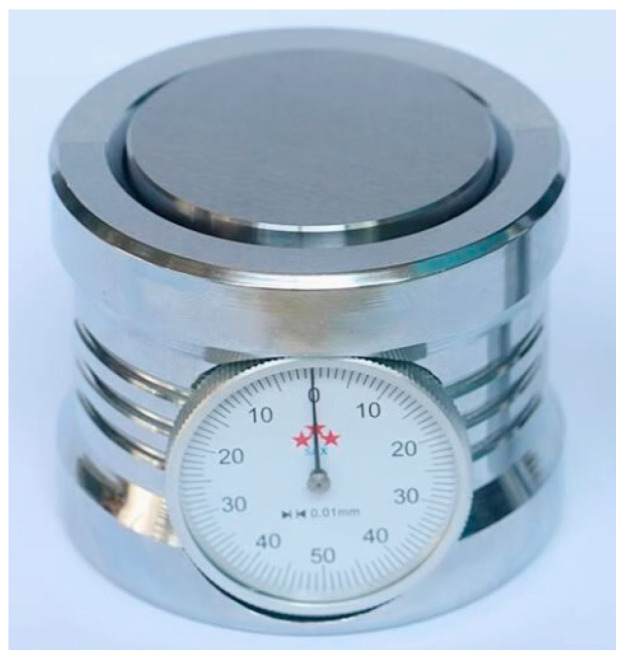
*Z*-axis setter.

The *Z*-axis setter is shown in Figure 5, with a height of approximately 50.00 ± 0.01 mm. It can be divided into various types such as circular, square, photoelectric, etc. Before aligning the tool, it is placed on the surface of the workpiece, the tool is controlled to contact the *Z*-axis setter, and its pointer is put to 0. The current coordinate value is recorded and the height set on the *Z*-axis from it is subtracted.

### 2.4. Dial Gauge or Micrometer Knife Method

**Figure 6:**
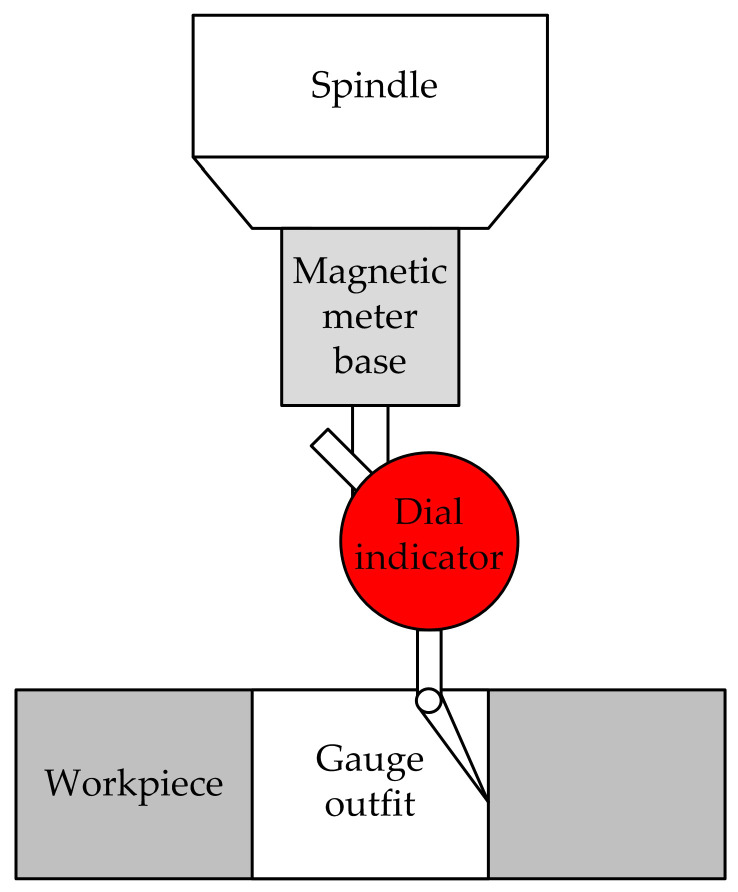
A dial gauge is used for tool alignment.

This method has complex operation steps and low efficiency, but high tool accuracy. Mainly used for aligning circular workpieces. When in use, the contact of the dial gauge contacts the circumferential surface of the part, and the spindle is slowly rotated by hand to make the contact of the dial gauge rotate along the circumferential surface of the part. The deviation of the dial indicator pointer is observed. By repeatedly adjusting the feed of the X and Y axes of the machine tool to ensure that the pointer’s runout is within the allowable range of tool alignment error, it can be determined that the center of the spindle is the center of the circular part [23]. A dial gauge is used for tool alignment is shown in Figure 6.

### 2.5. Contact Type Tool Setter

**Figure 7:**
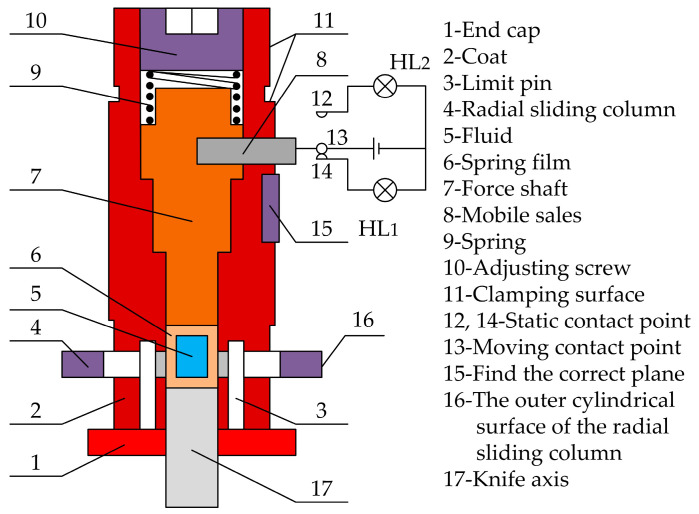
Numerical control comprehensive tool setting device for fluid transmission mechanism.

Dong et al. [24] designed a CNC-integrated tool setting device for fluid transmission mechanism, as shown in Figure 7. It has the advantages of simple operation, low cost, high cutting efficiency, and a wide range of applications. The operation method is as follows: The clamping surface 11 of the tool setting device is installed into the CNC machining tool holder and clamped. Its length is measured as the reference tool. Similarly, other tools are installed into the CNC machining tool holder and clamped. Their lengths are measured. The length of the reference tool from the length of other tools is subtracted to obtain the length compensation of other tools.

**Figure 8:**
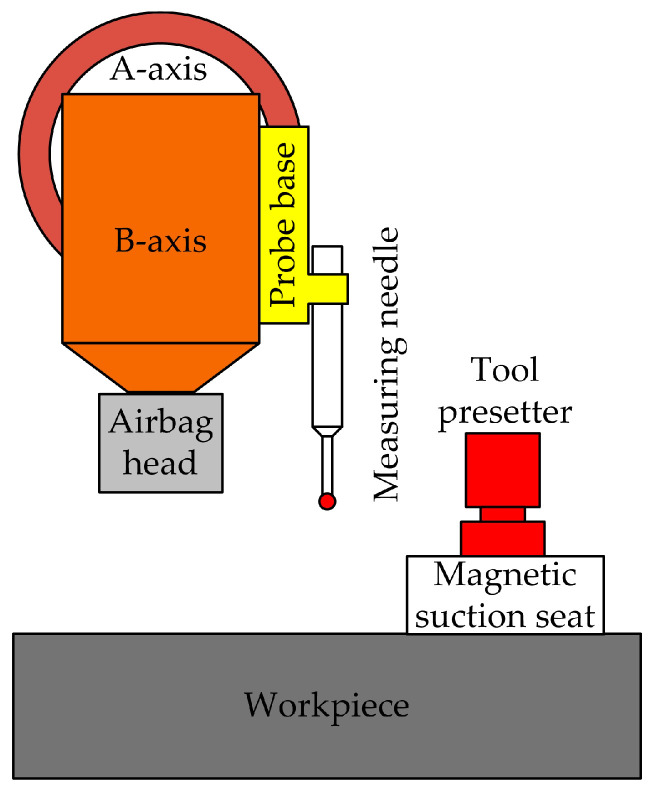
Automatic tool alignment system combining measuring head and tool alignment instrument.

Huang et al. [25] proposed an automatic tool alignment measurement method based on the combination of a measuring head and a tool alignment instrument and proposed an automatic tool alignment system that combines the measuring head and the tool alignment instrument, as shown in Figure 8. A measuring head is used to obtain the placement error and center point coordinates of the workpiece. The tool presetter is reused to determine the relative height relationship between the measuring head and airbag head in the tool presetting sequence. Compared to traditional manual knife alignment, this method has greatly improved speed. The repeatability accuracy of tool alignment in both X and Y directions reaches 30 μm, and in the Z direction it is 5 μm, which is increased by 40% and 50%, respectively, and has good engineering application value.

Operation method: The measuring head is moved downwards to trigger the tool setter, and the coordinate values of the measuring head are recorded. The airbag head is rotated 23° along the B-axis and moved downwards to trigger the tool alignment device. The coordinates of the airbag head are recorded and the height difference between the airbag head and the measuring head is determined. The B-axis is returned to zero, a measuring head is used to measure the four sides of the workpiece, and the placement error of the workpiece is calculated. The probe is moved downwards to trigger the probe. Finally, the coordinates of the center point of the workpiece can be calculated based on the coordinates of the airbag head. Thus, the tool measurement is completed.

**Figure 9:**
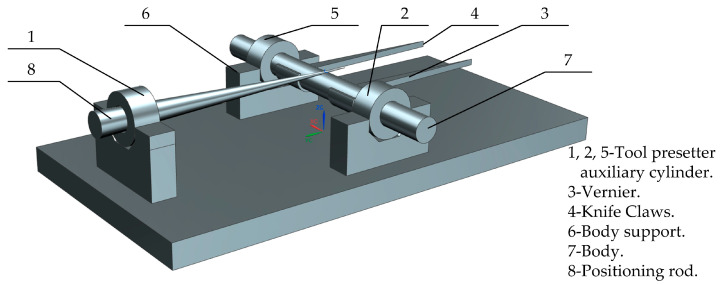
Schematic diagram of the structure of the universal lifting milling machine tool setter.

Ge et al. [26] designed a universal lifting table milling machine tool setter, as shown in Figure 9. It can achieve fast and accurate tool alignment, saving processing time. Operation method: The two tool claws (5) are adjusted to be slightly larger than the width value of the part to be processed, and they are fixed with fastening screws (3). The auxiliary cylinder of the tool setter is placed on the V-shaped bracket (7) of the fixture, and then the tool setter is placed on the auxiliary cylinder of the tool setter. The position of the cutterhead is adjusted to align with the axis of the fixture. The position of the fixture is adjusted so that the cutting surface is close to the cutter head. The blades are installed in sequence, tightly adhering to the mating surface. By following the above steps, the universal milling machine can complete the alignment of the shaft fork and obtain more accurate results.

**Figure 10:**
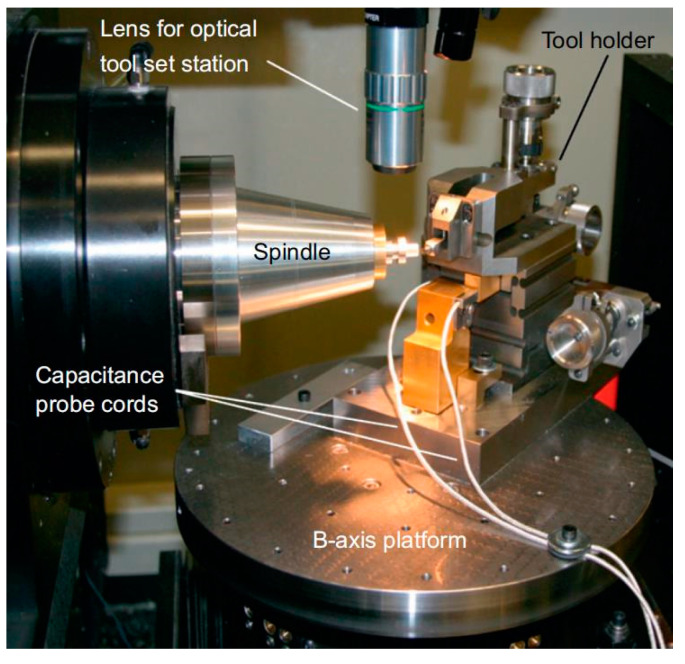
B-axis rotary table tool setting experimental device. Reprinted/adapted with permission from Ref. [27]. 2008, Elsevier.

Bono et al. [27] proposed a method for single-point tool alignment on a B-axis rotating disc on a precision lathe, and the experimental setup is shown in Figure 10. This method requires machining three grooves on the surface of the workpiece and setting the B-axis at three different angles. Then, the depth of each groove is measured and the measured values are used to calculate the tool offset. The experiment verified that this method can be used for precise tool alignment. It can be used with any size or geometric shape of single-point tools and can be used for extremely fragile single-point tool alignment without damaging the tool.

**Figure 11:**
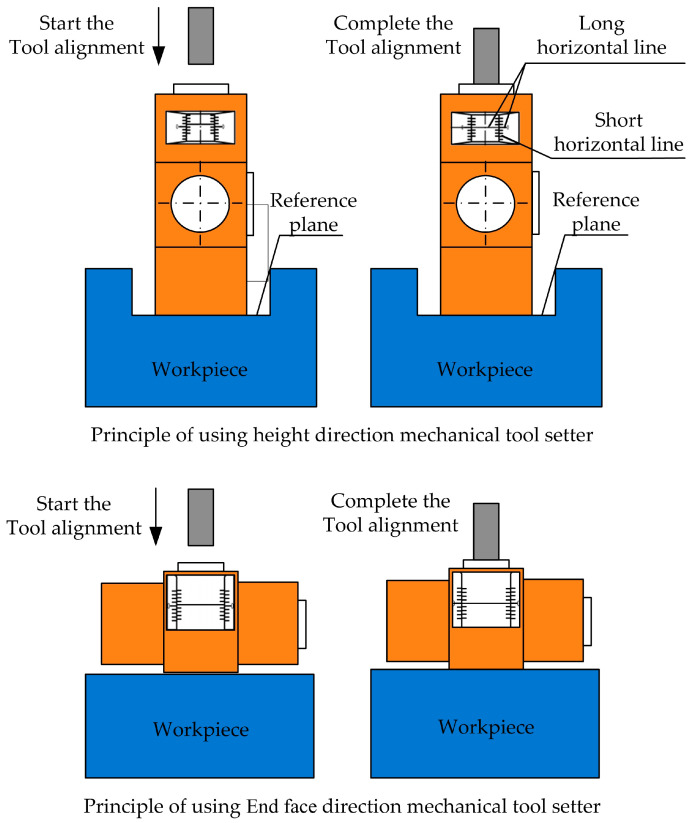
Principle of use of mechanical tool setter.

Wei et al. [28] designed a scale-based reading method, which adopts a combined mechanical tool setter that integrates height direction and the end face direction in appearance. (The principle of use of a mechanical tool setter is shown in Figure 11). The advantage is that the height direction can be used to measure reference surfaces such as holes and grooves, while the end direction can be used to measure flat surfaces. The combined tool setter not only has a wide range of applications but also has low cost and accurate positioning. The operation method is as follows:

Height direction: The tool setter is placed on the reference surface of the part groove and the tool is moved to the upper end of the top surface of the measuring column. The handwheel *Z*-axis is rotated to make the tool contact the top surface of measurement. By fine-tuning the *Z*-axis of the handwheel, the zero-point scale position of the measuring column is aligned perfectly with the zero-point scale position of the base body. The handwheel operation is stopped, the current coordinate value is recorded, the machine tool Z-value is inputted, and the height of the tool presetter is subtracted to complete the height tool presetting measurement.

End face direction: The tool setter is placed on the reference surface and the tool is moved to the upper end of the top surface of the measuring column. The *Z*-axis of the handwheel is rotated to move the tool into contact with the top surface of the measuring column. By fine-tuning the *Z*-axis of the handwheel, the zero-point scale position of the measuring column is aligned with the zero-point scale position of the base body, and then the current coordinate value is recorded. The Z-value of the machine tool is entered and the G54 coordinate origin of the machine tool is input to complete the end face direction tool alignment measurement.

**Figure 12:**
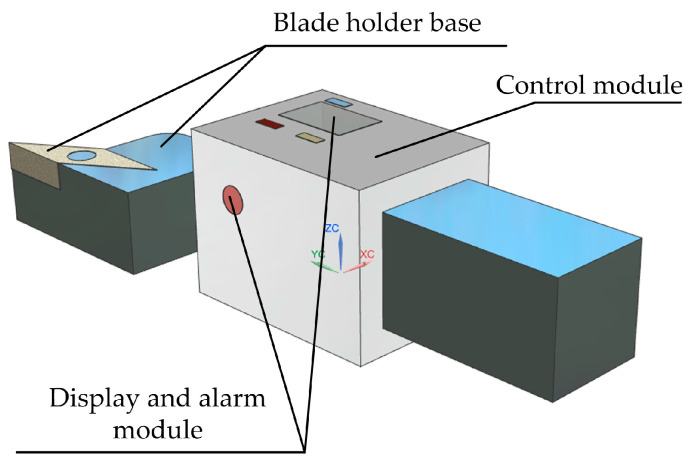
Turning electronic tool setting device.

Yang et al. [29] researched and developed a turning-type electronic tool setting device, as shown in Figure 12. This device improves tool alignment speed and accuracy, avoiding damage to the machined surface and protecting the cutting edge of the tool. The device includes a mechanical module, a control module, and a display and alarm module, providing real-time and accurate tool alignment information through sound, light, and digital display. Operation method: The power button is turned on to bring the tool into contact with the workpiece. The LED light will turn on and the buzzer will beep briefly, indicating that the cutting edge of the tool has already made contact with the workpiece. When the tool continues to move to the ideal tool depth, the buzzer will beep three times, indicating that the tool has entered the preset error range and cannot be fed. The LCD screen is observed, the absolute gap value from the value displayed on the LCD screen is subtracted, the gap compensation on the tool alignment interface is input, and the tool alignment operation is completed.

**Figure 13:**
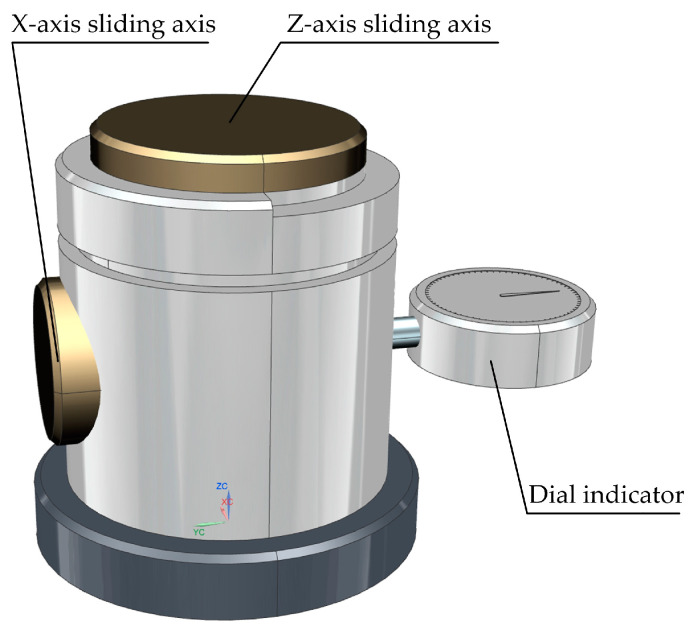
Mechanical transmission type CNC lathe tool setter.

Wu et al. [30] designed a mechanical transmission-type CNC lathe tool setter, as shown in Figure 13. Using mechanical movement to transmit the displacement of the tool to the dial gauge, and using a relative comparison method to measure the compensation values in the *X-* and *Z*-axis directions of the tool, thus achieving tool alignment.

Operation method: Combined with the trial cutting and tool alignment method, utilizing the convenient, accurate, and high-precision characteristics of the dial gauge reading, the mechanical movement of the tool in the *X-* and *Z*-axis directions is displayed through the dial gauge, and compared with the reference size to measure the *X* and *Z* compensation values of the tool, thus achieving tool alignment.

**Figure 14:**
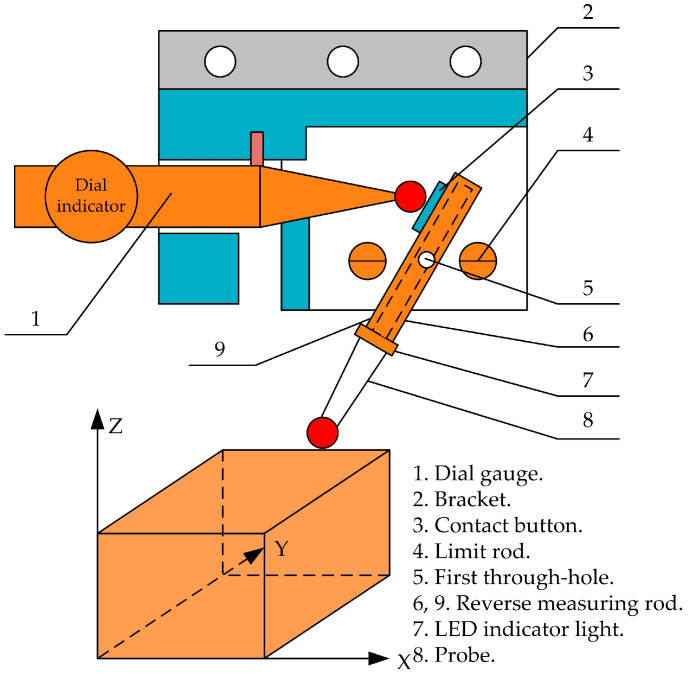
Contact electronic knife setting device.

Luo et al. [31] designed a contact-type electronic knife setting device using the principle of lever and laser emitter, as shown in Figure 14. This device is easy to use and reliable, improving the accuracy and efficiency of tool alignment. Operation method: The dial gauge is assembled, the measuring rod and bracket are reversed, and then the device is installed inside the machine tool spindle. The spindle of the machine tool is rotated, the installation of the fixture is checked, and the installation of various components of the device is adjusted. The machine tool is moved to make contact between the reverse measuring rod and the workpiece, and the LED light is checked to determine if the contact is effective. When the light is on, it indicates that the calibration is effective. When the light goes out, the calibration is ineffective. The other coordinate axes of the machine tool are adjusted until the LED light is on and then the calibration operation is performed. 

## 3. Non-Contact Tool Alignment Method

### 3.1. Image Based Knife Technique

**Figure 15:**
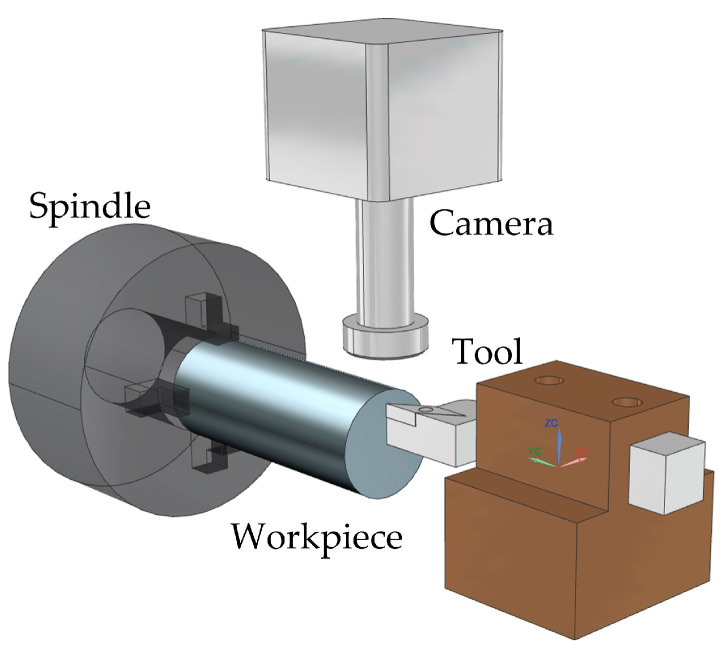
Quantifiable B rotation axis tool alignment experimental device.

Duong et al. [32] proposed a quantifiable B-axis tool alignment method based on the principle of determining the center of the circle using 3-point coordinates. The experimental setup is shown in Figure 15. He adopted a non-contact image recognition method for precise tool alignment, avoiding damage to diamond-cutting tools during the alignment process and solving the problem of an inconsistent tool tip field of view and machine tool coordinates. The machining experiment of the Fresnel lens mold was used to verify the tool alignment accuracy, and the arc radius of the sharp corner in the groove was detected to determine the tool alignment error. The experimental results show that the quantifiable tool setting method has a tool setting error of 0.8 μm. This tool setting method is suitable for ultra-precision cutting, and the accuracy of the tool setting can be predicted and controlled. 

Tool alignment operation: The coordinate system between the image and the machine tool is calibrated, and the relative position of the tool tip in the Z direction at different angles is measured. The blade tip is moved to the camera field of view for recognition. The relative position of the cutting edge can be calculated by adding the relative position on the image to the relative position on the *Z*-axis of the CNC system to determine the distance between the cutting edge and the center of the B-axis. By displaying the distance through the image, it is easy to adjust the tip of the knife to the target position. Thus, the knife is completed.

**Figure 16:**
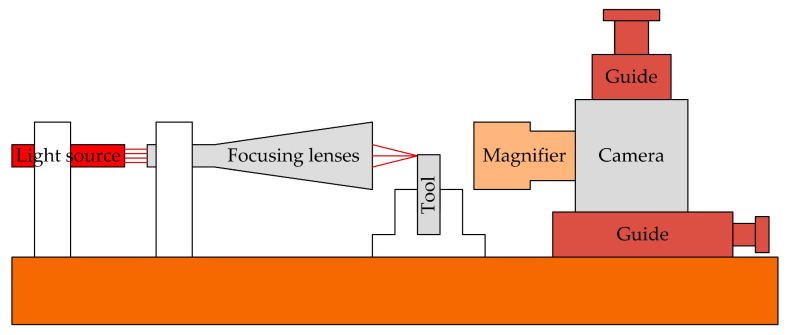
Digital holographic knife alignment device.

Cheng et al. [33] proposed a non-contact knife alignment method based on digital holographic in-situ imaging, and the knife alignment device is shown in Figure 16. For the imaging problem of micro-milling cutters, an evaluation function based on wavelet transform is introduced in the progressive search process, and the image is accurately and optimally reconstructed through an autofocus algorithm. Then, the zero-order terms are eliminated and the images are conjugated through self-snake diffusion. The tool contour is extracted using a Sobel operator and the tool diameter is measured using different methods. The measurement results indicate that holographic imaging can meet the requirements of dynamic measurement of micro-milling cutter positioning, and also provide an important basis for the accuracy of tool alignment, improving the precision of tool alignment.

**Figure 17:**
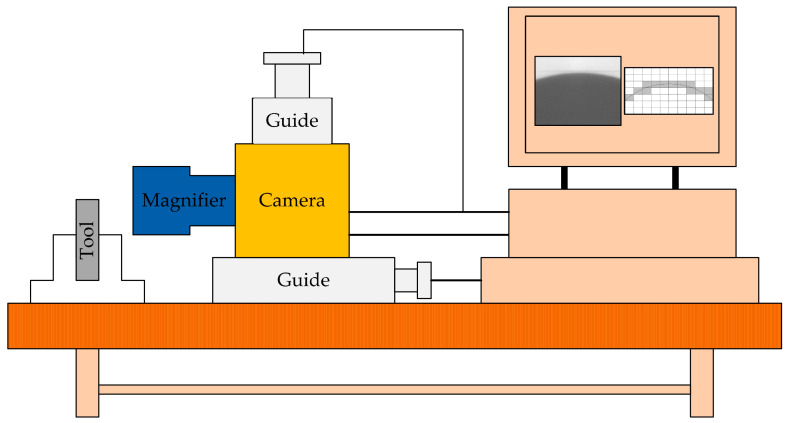
Non-contact precision tool alignment system.

Chao et al. [34] developed and constructed a non-contact precision tool alignment system using edge detection image processing and sub-pixel segmentation techniques, combined with a precision turning machine numerical control controller, as shown in Figure 17. Research has found that better results can be obtained when evaluating the length of the entire 6–8 pixel range. The results show that the developed tool alignment system can be applied to tools of different shapes, and the positioning error range is ±0.1 µm.

Tool alignment operation: After capturing the tool image with a CCD camera, a digital image function is generated based on the grayscale of the image sampling points on the CCD sensor (including information about the tool and various noises caused by fragments, reflections, diffraction, and background scattering), and a median filter is used for noise reduction. Using image analysis techniques such as Laplacian, Sobel, Roberts, 8Canny, and Zernike operators for edge detection, tool contours are extracted from the image to determine the tip position and its shape error. Using the least squares method to average the noise, the linear fitting line can be used for sub-pixel measurement. Finally, the knife alignment is completed.

**Figure 18:**
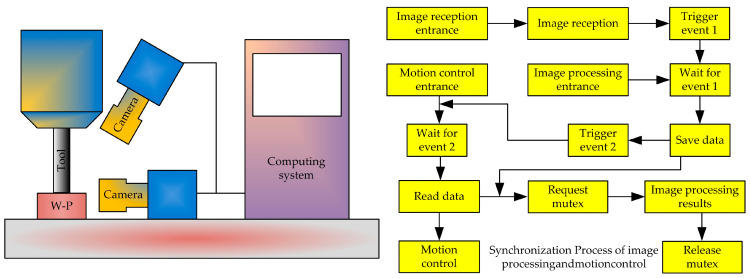
CNC machine tool motion control system.

Wang et al. [35] proposed a CNC machine tool motion control system based on image processing. The overall structure of the system is shown in Figure 18. It mainly consists of three parts: image acquisition, image analysis, and motion control. The simulation and testing results show that the proposed system not only has a fast control speed but also ensures the overall design of the control accuracy system.

Tool alignment operation: This includes two steps: zero-point return and quick positioning. Firstly, the center coordinates of the workpiece obtained through image analysis are used to determine the tool alignment point coordinates, and the motion control card drives the motor to rotate based on the coordinate information to achieve horizontal alignment of the tool. Then, based on the tool distance obtained through image processing, the *Z*-axis descent distance of the machine tool is controlled, and the descent of the tool is monitored in real-time to achieve vertical alignment.

**Figure 19:**
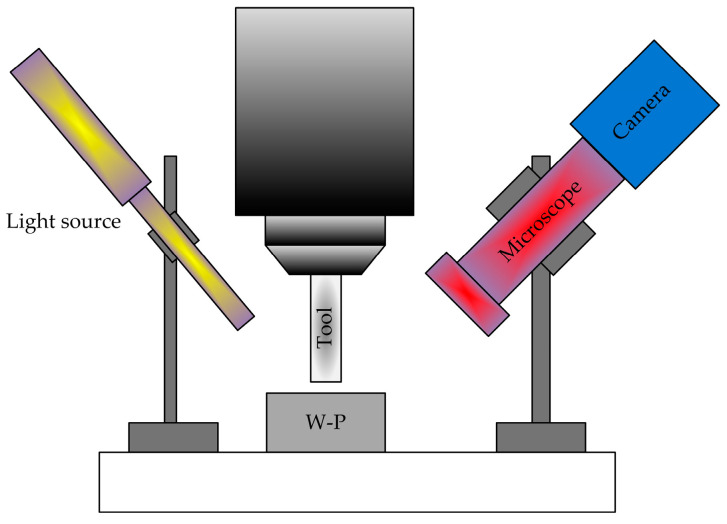
Experimental tool setting device.

Zhao et al. [36] and Zheng et al. [37] proposed a method for the automatic tool alignment of micro-cutting tools based on microscopy and image processing. The knife-setting device is shown in Figure 19. Firstly, real-time images of the cutting area are obtained through a microscope and CCD camera, followed by image processing. The gap distance between the tool and the workpiece is calculated, and then it is sent to the machine motion control card to make the tool move the corresponding distance to contact the surface of the workpiece. Then, these steps are repeated until the gap is zero, and finally the tool alignment is completed with an accuracy of 6 μm.

**Figure 20:**
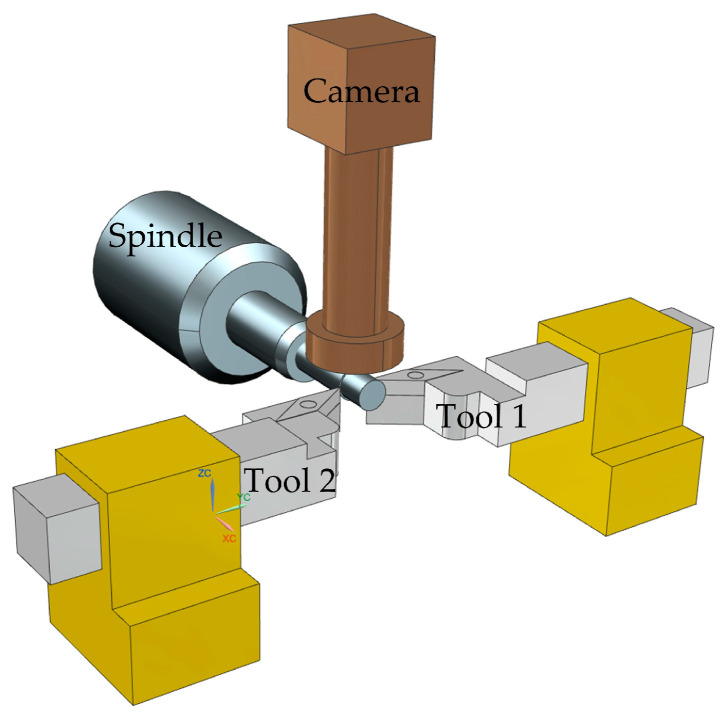
Two-dimensional tool gap detection experimental testing device.

Yu et al. [38] designed a two-dimensional tool gap detection device for micro lathe tool alignment, as shown in Figure 20. This device can quickly and effectively identify and detect the tool clearance of micro lathes, with a system detection accuracy of up to 15 μm. At the same time as the experiment, the correctness of the tool gap detection algorithm was verified, which can be used for precision tool alignment of micro lathes.

Tool alignment operation: The offset between the tool and the workpiece is manually adjusted to align the tool with the workpiece end face. Extract the workpiece and tool from the image to be inspected obtained by the optical system. And perform positioning of the workpiece rotation center and tool tip. Adjust the offset between the tool and the workpiece. Adopting regional growth method for workpiece area positioning. Then extract the contour of the workpiece and perform minimum circumcircle fitting. Determine the position of the workpiece and set it as the reference position for measuring the tool clearance. Calculate gradient images, image segmentation, and refinement. Use Hough transform to extract fitting lines, and finally complete tool tip positioning to determine the tool clearance, thus completing tool alignment.

**Figure 21:**
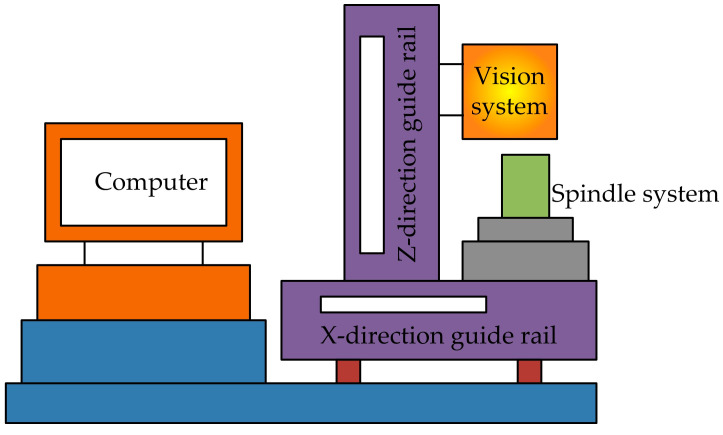
A schematic diagram of the composition of the electronic camera-type tool setter.

Liu et al. [39] studied an electronic camera-based tool setter, as shown in Figure 21. The measurement principle and system structure were analyzed, and the automatic focusing and calibration methods of the system were discussed. It has the characteristics of high precision and a high degree of automation, providing a guarantee for the high-precision machining of CNC machine tools. The experimental results show that the repeatability accuracy of the cutting point measurement of the electronic camera tool setter reaches ±2 μm.

Tool alignment operation: A fast sub-pixel edge detection method is adopted for measuring images. The standard Sobel operator is used for rough localization of edge points. The pixel-level precision position of edge points and the direction of edges is determined. The pixels are expanded along the edge direction of the edge points to obtain a pixel grayscale value vector with a length of 6. The vector is substituted into the formula obtained using the least squares curve fitting method to determine the precise position of the edge points. Thus, sub-pixel-level edge localization accuracy can be achieved. The obtained sub-pixel precision edge points are used to fit the parameters of the tool tip. Then, the knife alignment is completed.

### 3.2. Laser Diffraction/Direct Cutting Method

**Figure 22:**
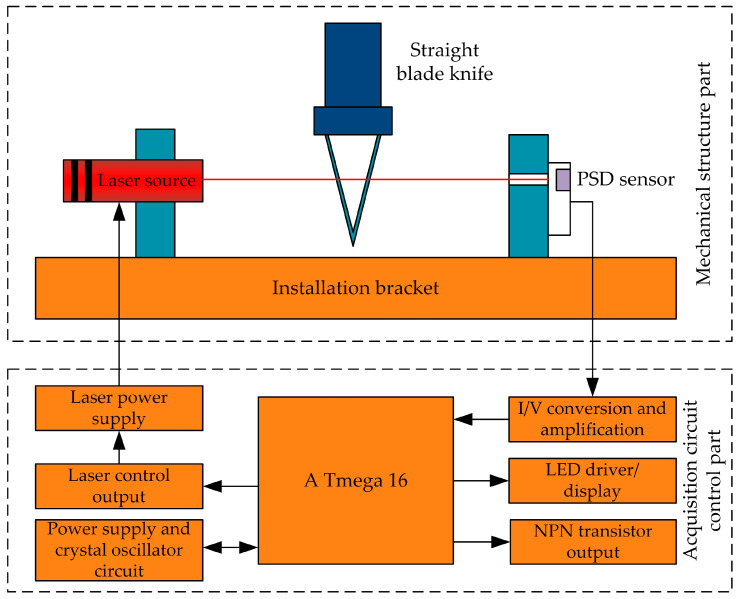
Experimental system diagram of laser alignment with a straight blade knife.

Yu et al. [40] proposed a laser alignment method for ultrasonic, straight-edge knives based on the parallel laser occlusion theory, and the alignment system is shown in Figure 22. A one-dimensional PSD measurement and calculation model for obtaining the angle of an ultrasonic straight blade was derived and established. The influence of different tool section width parameters on angle detection sensitivity was analyzed. The design of mechanical and electrical solutions was conducted, as well as acquisition and control programs. The concept of “reference value for zero position angle” was proposed. Finally, a laser alignment instrument experimental system was constructed. Performance testing and analysis research on zero position angles was conducted. The results indicate that the developed laser alignment instrument for straight-edged knives has good zero position angle acquisition accuracy.

Tool instruction: The laser source is turned on and the tool is controlled to start rotating by outputting a signal through a PNP transistor. The timer is started, the AD acquisition and storage cycle is entered, and the cycle is ended after exceeding the set time Δt. The “zero position angle reference value” is calculated and obtained. The high-speed acquisition of one-dimensional PSD output signals is performed, and after processing such as sliding average filtering, the data enter the AD acquisition and display cycle. If the “zero position angle reference value” is reached, the loop ends. Simultaneously the zero-position signal is output and the tool rotation is turned off to complete the tool alignment.

**Figure 23:**
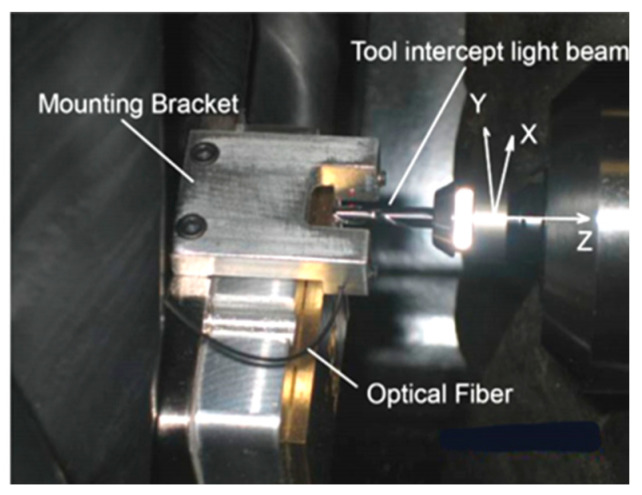
Fiber optic shielding tool setting device. Reprinted with permission from Ref. [41]. 2017, Elsevier.

Liu et al. [41] studied a non-contact automatic tool setter for micro-milling machines, which can greatly shorten the tool setting time. The non-contact knife setting device with fiber optic obstruction is shown in Figure 23. The focus of the research was to address tool alignment in the *Z*-axis. Keyence’s fiber optic FS-V30M sensor was used, Which was equipped with light-emitting elements and receiving sensors. The sensor emits a beam of light from its light-emitting element. At the receiving end, the sensor measures the change in light intensity caused by the micro tool passing through the optical axis. The smallest detectable object is 5 mm, suitable for micro tool detection (5–500 mm). The sensor detects the position of the micro tool by measuring the change in light intensity when the tool passes through the emitted beam. Xinyu Liu also proposed a new edge detection and search algorithm. This algorithm is implemented in the controller of a CNC machine tool. This algorithm achieved 0.6 mm repeatability on different tools. A precision of 2 mm was achieved in actual microfabrication devices. Knife alignment takes about 10 s, with a reduction of 80–90% from manual alignment.

Knife alignment operation: A probe is used to measure the displacement in the Z direction between the edge of the beam and the workpiece reference. Two encoder readings are recorded when the probe is located at the edge of the beam and when the probe contacts the reference surface separately. The difference is displacement D. This displacement is fixed on a given workpiece, and is used to offset subsequent tool length changes under the same fixture settings. The probe is removed from the spindle and a new tool is installed. Bring the new tool tip to the edge of the beam and record the reading of the corresponding encoder on the *Z*-axis. Finally, by offsetting, the *Z*-axis of the tool will be set to zero.

**Figure 24:**
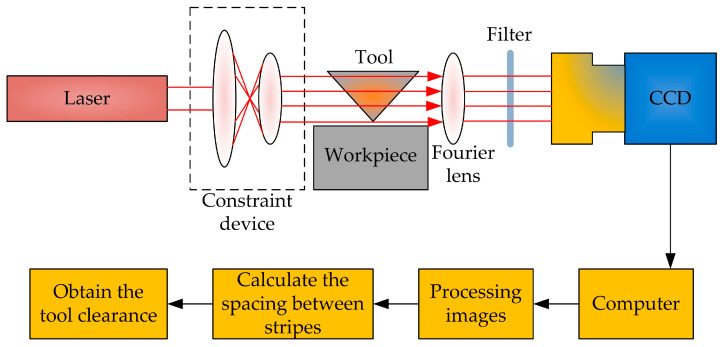
Laser diffraction knife experiment device.

Zhang et al. [42] proposed a diamond-cutting tool alignment method based on the principle of laser diffraction, and the experimental setup is shown in Figure 24. Alignment operation: The alignment prototype is installed and fixed in the appropriate position, and the height of the alignment device is adjusted to ensure that the laser, alignment spacing, and CCD image plane are in the same reference plane. The detection mechanism is rotated to the reference plane by rotating the deflection stage of the optical axis. When the laser passes through the gap between the tool and the workpiece surface, diffraction occurs, forming diffraction fringes. The Fourier lens converges the diffraction fringes and forms an image in the CCD. The CCD is used as a visual sensor to receive diffraction fringes and transmit diffraction fringe information to a computer. After image processing and detection using computer image processing software, the distance between the cutting edges is obtained, and finally, laser diffraction cutting is completed.

**Figure 25:**
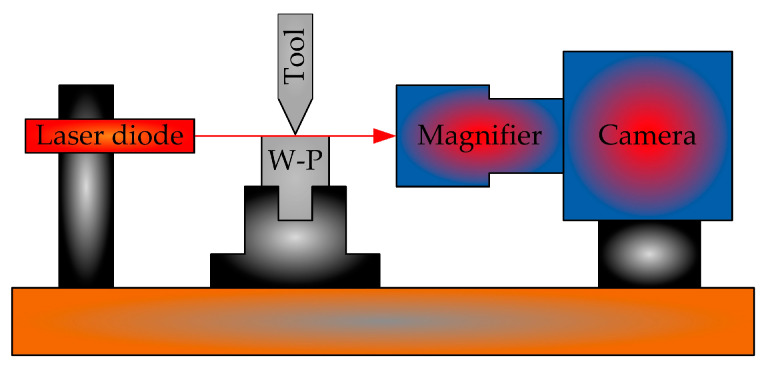
Experimental setup for measuring the diameter of micro-cutting tools.

Khajornrungruang et al. [43] from Japan proposed a non-contact online measurement tool method using laser diffraction and optical systems. The diameter of micro-cutting tools was used for non-contact optical measurement of sub-micron accuracy by subtracting the transparent laser component from the laser mixture distribution between the transparent laser component and the diffracted laser component. Only the laser components that enhance diffraction patterns are obtained to enhance the characteristics of the diffraction pattern. Experimental measurements have verified the feasibility of this method. In addition, a tool diameter measuring device that can be installed on machine tools during high-speed rotation has been developed for preliminary measurement of micro drill bits. The results indicate that the proposed laser diffraction method can measure the diameter of micro-rotating cutting tools during actual high-speed machining. Meng Xu et al. [44] proposed a method for compensating tool alignment errors using a laser imaging device. This method can directly detect the actual tool position and calculate the tool center point coordinates on the machine coordinate system. It can perform non-contact measurements and the machining of small and complex-shaped cutting tools. It is possible to measure and calculate the initial setting error of the tool center point relative to the rotation axis center without any trial cutting. In order to improve compensation performance, the laser imaging device is calibrated on ultra-precision machine tools. By modifying the NC program, it is ultimately possible to compensate for tool alignment errors. The experiment verified that the proposed compensation method is feasible and effective. The experimental setup is shown in Figure 25.

**Figure 26:**
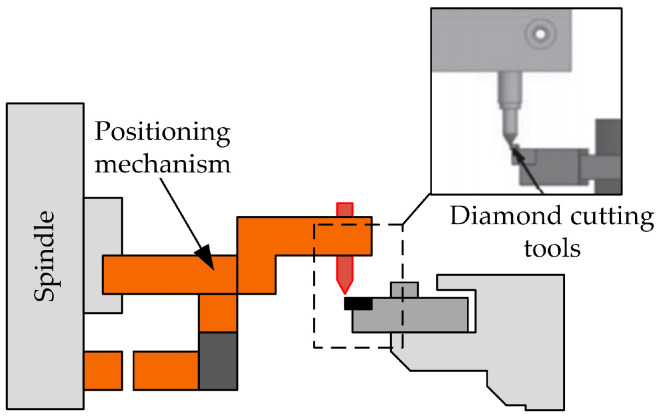
Mechanical LVDT tool setting device.

Song et al. [45] used a combination of coarse and fine positioning through an LVDT knife alignment device and an optical knife alignment device. The principle is shown in Figure 26. The center height, tip radius, and tool centering coordinates of the tool are quickly and accurately measured. The repeated tool alignment accuracy reaches 1 μm, achieving precise tool alignment. The tool setting device is applied to a self-developed three-axis diamond lathe SGDT350 machine tool. The ultra-precision machining of convex spherical aluminum parts with a diameter of 75 mm and a top ball diameter of 250 mm is achieved. Its surface accuracy PV reaches 0.24 μm. This further proves the accuracy of the tool setting device and the effectiveness of the method, while significantly improving the tool setting efficiency.

### 3.3. Cutting Method Based on Discharge Principle

**Figure 27:**
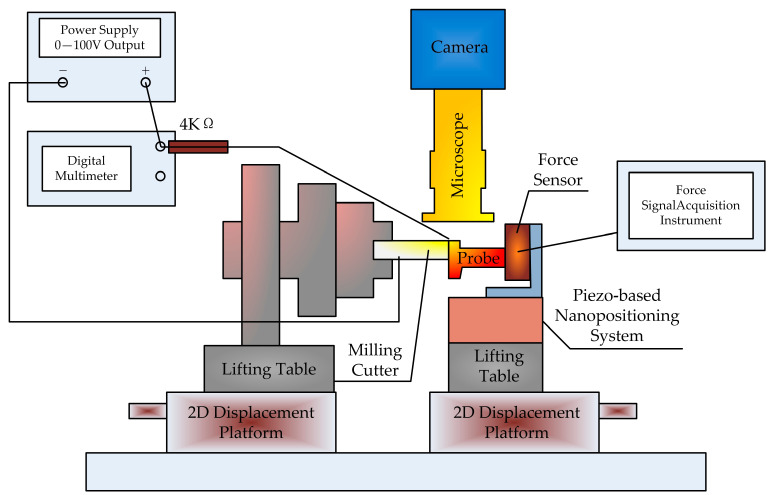
A diagram of the cutting experiment device based on the discharge principle.

Xu et al. [46] proposed a sub micron level tool alignment method based on the principle of micro gap discharge. The accuracy of this method is ±2 µm, and its tool setting device is shown in Figure 27. He conducted experimental verification on the feasibility and accuracy of the knife pairing device. The results show that when the gap between the tool and the tool probe is 2 µm, the breakdown voltage is about 30 V. With the increase in tool wear, the breakdown voltage shows an upward trend and can be used for tool wear monitoring. This method also has the advantages of no contact, a simple structure, and a low cost, and it is suitable for the alignment of micro-cutting tools.

Tool alignment operation: A preset voltage is applied to the tool and probe with a variable voltage source. The current is connected through the amplification circuit and filtering circuit to the signal recognition circuit after processing. The tool is moved to the probe until breakdown occurs. When the breakdown current is greater than the pre-set threshold current, the signal recognized by the signal recognition circuit is used as a signal of successful tool alignment. A successful tool alignment indication is sent through the signal-indicating circuit, and the signal is uploaded. The CNC system records the coordinate value at this time and then subtracts the discharge gap (the coordinate value of the origin of the cutting edge in that direction).

**Figure 28:**
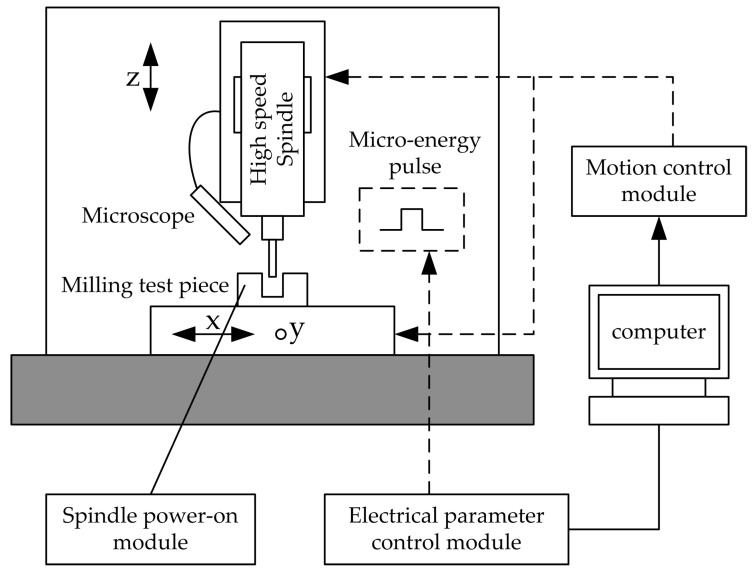
Schematic diagram of the cutting system based on discharge sensing [42].

Hu et al. [47] proposed a precision automatic tool alignment technology for micro-milling cutters based on the principle of micro energy pulse discharge sensing and verified its feasibility through experiments. The principle of this technology is to achieve tool alignment by judging the discharge state of the gap when the workpiece and the tool slowly approach each other. During the discharge sensing operation, the micro-milling cutter slowly approaches the workpiece through the machine tool’s numerical control system. When the gap between the micro-milling cutter and the workpiece is smaller than the discharge gap, discharge occurs, and the voltage or current of the gap changes, and the sensing circuit captures the change signal. Feedback signals are provided to the machine tool to record real-time motion coordinates, thereby achieving workpiece positioning. The schematic diagram of tool alignment based on the discharge sensing principle is shown in Figure 28.

**Figure 29:**
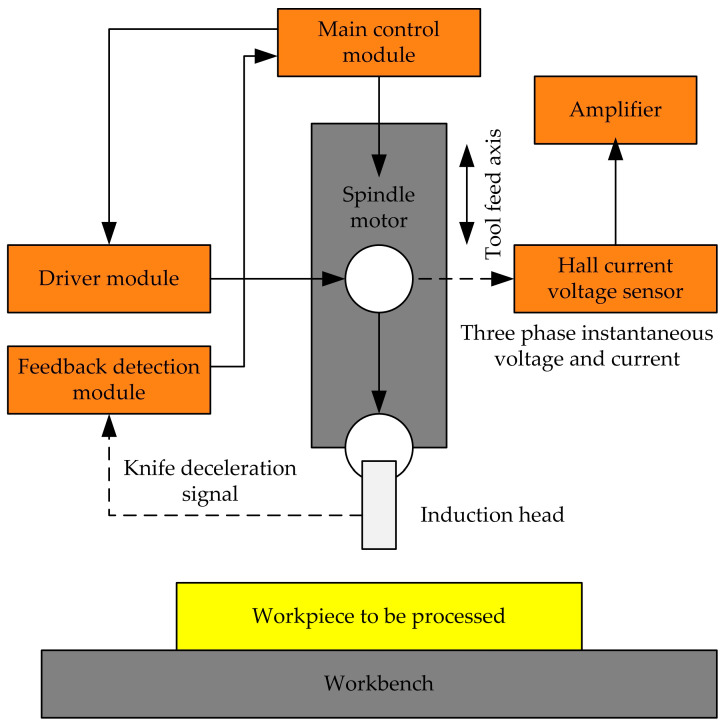
Schematic diagram of the tool alignment process of the automatic tool alignment device.

Fan et al. [48] proposed a two-stage tool alignment method that combines electrical detection and instantaneous power detection. The schematic diagram of the automatic tool alignment process is shown in Figure 29. A PARK transformation model based on instantaneous power detection was established for the post-stage tool setting that determines the accuracy of the tool setting. This model was used to sequentially detect two sets of three-phase instantaneous active power values at corresponding sampling points separated by two half cycles of the power grid. A data judgment model is provided based on the information weighting method, and sequentially the detected two sets of data are input into the model for comparison and judgment. The data that meet the conditions are counted, and the tool alignment is considered complete when the count reaches the set value. The experimental results show that the tool setting accuracy of this method is at least twice as high as other methods that use the detection of spindle motor power. It has the characteristics of simple operation, high reliability, and high accuracy.

Tool alignment operation: In this project, a two-stage knife alignment method is used to achieve high-precision automatic knife alignment. For the front-end feed module, the main control system sends a signal to the spindle motor to start tool alignment, and the tool is fed at a higher speed. When the sensing head of the feedback detection module contacts the ground workpiece, the preceding deceleration signal sensing module immediately generates a deceleration signal. At the same time, the sensing head retracts back to the back of the tool. For the later stage tool setting module, the grinding CNC system applies a minimum speed feed signal to the tool feed axis to feed it with a small resolution. Simultaneously, the three-phase current and voltage of the tool spindle motor is detected in real-time, and the instantaneous three-phase active power is calculated based on this. At the moment when the grinding tool comes into contact with the workpiece being ground, the system immediately applies a stop feed command to the tool feed axis based on the change in power. At this point, the stop position of the tool feed axis is the tool alignment position.

### 3.4. Visual-Based Knife Alignment Method

**Figure 30:**
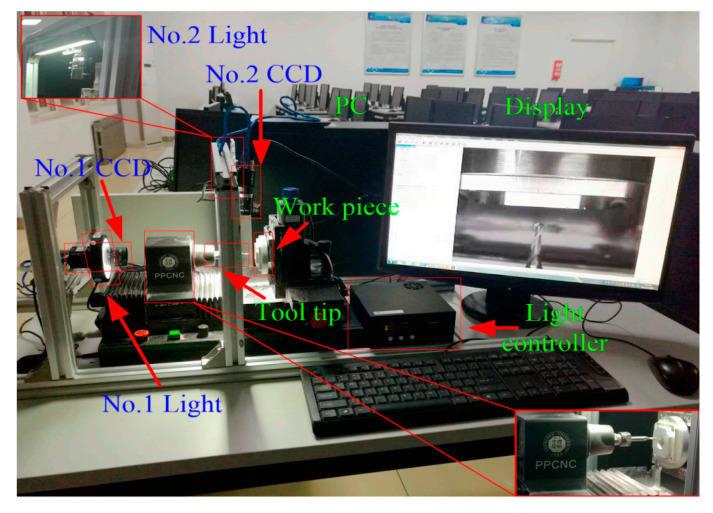
Automatic knife setting experimental device system [44].

Hou. et al. [49] proposed an automatic tool alignment method for CNC machine tools based on computer vision. On the basis of the traditional visual system calibration theory, a high-efficiency and high-precision CNC machine tool visual automatic setting system was developed. The experimental setup is shown in Figure 30. System calibration and computer vision tool measurement experiments are completed. The coordinate mapping relationship between the image and the CNC machine tool, the tool position marking points on the workpiece, and the tool tip is calculated. Visual system performance testing and system calibration experiments are conducted. The experimental results show that the visual-based knife alignment method can achieve image processing with a time consumption of 128 ms, and its efficiency is nearly 100 times that of traditional knife alignment schemes. The cutting accuracy and measurement accuracy are less than 1 μm.

**Figure 31:**
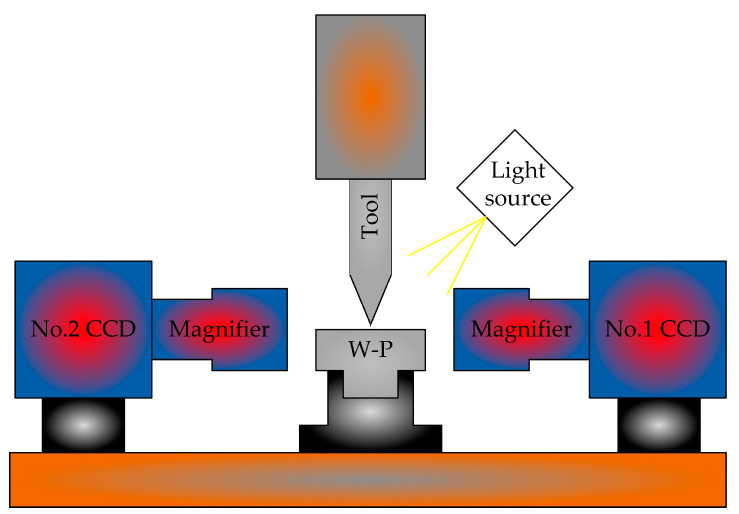
Machine vision automatic knife alignment experimental device.

Sun et al. [50] designed a three-dimensional tool gap detection device for machining small-sized workpieces. The principle is shown in Figure 31. Orthogonal arrangement is used to construct an image measurement optical system, and the high-precision characteristics of the machine tool itself are utlizied to accurately calibrate the optical system. The effects of uneven environmental lighting and target reflection are eliminated through top hat transformation, linear deepening, image segmentation, and region growing. The complete extraction of the target is achieved. The sub-pixel edges of targets are extracted through Zernike moments, straight lines are detected through Hough transform, the Z-direction distance to locate tool cutting points is calculated, and a CNC machine tool clearance detection system is established. Small displacements on the experimental platform for system validation are applied. The experimental results show that the detection system can quickly and effectively recognize and detect the tool clearance of CNC machine tools, with a detection accuracy of ±2 µm and an image processing time consumption of 2.26 s. The accuracy of the system meets the requirements of the modern manufacturing industry.

## 4. International Major Knife Setting Products

### 4.1. International Contact Type Tool Setter

**Figure 32:**
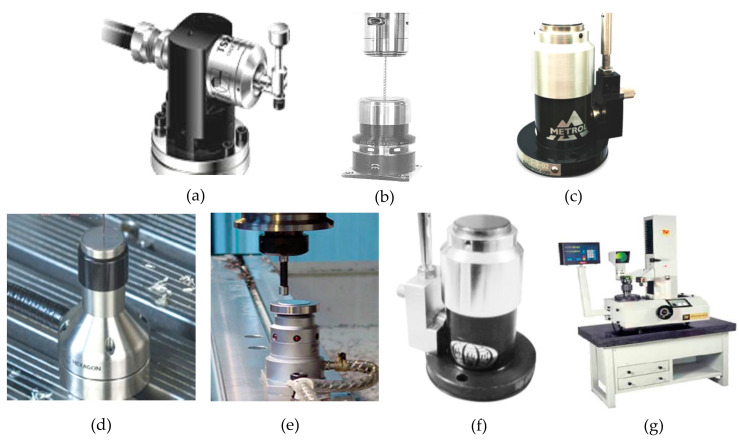
Internationally renowned non-contact tool setters. (**a**) TS27R tool setter from Renishaw; (**b**) Z-NANO tool setter from German company Polon; (**c**) TM26D knife alignment device from Japan’s Meidelong; (**d**) TS35.20 tool setter from Hexacon, Sweden; (**e**) ETC-3L tool setter in Harbin; (**f**) GZ-66 tool setter from Shenzhen Hainachuan; and (**g**) DTYⅡ540 tool setter from Tianmen Precision Machinery.

The development of contact type tool presetters has tended towards productization. Internationally renowned tool alignment brands include the following: The Renishaw TS27R tool presetter from the UK (as shown in Figure 32a). The Polon Z-NANO knife alignment device from Germany (as shown in Figure 32b). The Meidelong TM26D knife alignment device from Japan (as shown in Figure 32c). Sweden’s Hexacon TS35.20 tool setter (shown in Figure 32d). Harbin’s Pioneer Electromechanical ETC-3L tool setter (shown in Figure 32e). The GZ series tool setter (as shown in Figure 32f) from Shenzhen Hainachuan Electromechanical Co., Ltd. The repeated measurement accuracy of the latter contact type tool setter is around 1 μm, and the tool measurement range is above 0.5 mm. It can also detect tool breakage. The DTYⅡ1540 tool setter produced by Tianmen Precision Machinery Co., Ltd. in Tianjin (as shown in Figure 32g) has a measurement accuracy of 1 μm. In terms of functionality, this device can not only detect the tool spacing but also the radial and axial dimensions of the cutting edge of the tool. However, it still cannot meet the requirements for the high-precision measurement of tool spacing in ultra-precision machining.

### 4.2. International Non-Contact Tool Setter

**Figure 33:**
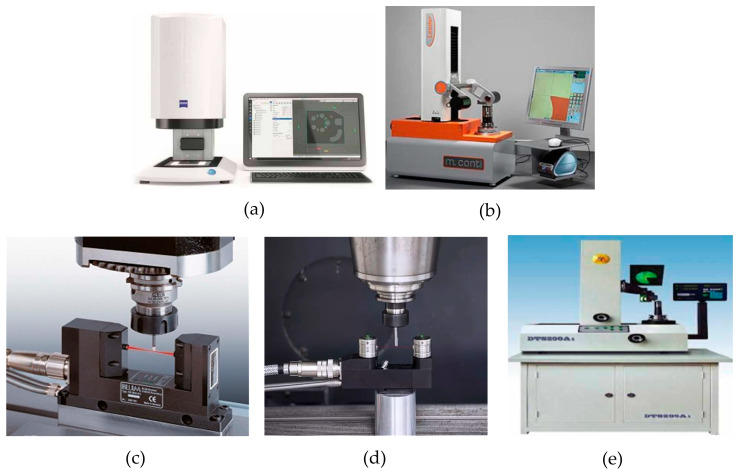
Well-known non-contact tool presetters, at home and abroad. (**a**) German Zoller electronic camera tool presetter; (**b**) Lead series electronic camera tool setter; (**c**) Bolong Company Laser Tool Alignment Instrument; (**d**) Renishaw NC4 laser tool setter; and (**e**) Tianjin University electronic camera tool pre-adjustment measuring instrument.

In the international market, the Smile400/4-plot2.0 tool presetting measuring instrument produced by ZOLLER company in Germany is renowned. It can achieve a display resolution of 1 μm and a repeated measurement accuracy of 1 μm, as shown in Figure 33a. The Leader series electronic camera tool setter developed by Company M.CONTI in Italy is also well known. The maximum measured diameter is 320/520 mm, and the repeated measurement accuracy reaches 1 μm, as shown in Figure 33b. The NT series laser tool setter developed by Blum Novotet Gmbh has a repeated measurement accuracy of 1 μm, and it can measure tools with a minimum diameter of 0.2 mm, as shown in Figure 33c. The NC4 non-contact laser tool setter launched by Renishaw in the UK has a repeated measurement accuracy of 1 μm, and it can measure cutting tools with a minimum diameter of 0.2 mm. By utilizing Renishaw’s microporous technology, it is also possible to provide uninterrupted protection for the system, as shown in Figure 33d. Tianjin University developed the first domestically developed electronic camera-based tool pre-adjustment measuring instrument with independent intellectual property rights. Its display accuracy reaches 1 μm, and the repeatability measurement accuracy reaches 2 μm, as shown in Figure 33e.

## 5. Conclusions

With the development of science and technology, various methods of tool alignment have emerged. Although they have differences, their purpose is consistent, which is to achieve precise tool alignment. This article introduces the commonly used tool alignment methods and steps for machine tools. The contact type tool setting method and non-contact type tool setting method were analyzed, as well as the mature tool setting instrument products mainly used internationally. It can be found from the review that contact-type tool presetters have moved in the direction of productization. Although products with the same performance indicators are priced higher in countries such as the United States, Japan, Germany, the United Kingdom, Italy, and Sweden than in China, the most commonly used tool presetters by enterprises are still big brands, which have more mature technology compared to other brands. For non-contact tool presetters, China mainly uses electronic camera tool presetters.

Although there has been some progress in tool alignment technology, it still faces some challenges. Some examples are as follows. Environmental adaptability: Cutting technology needs to maintain stability and reliability in different processing environments. Cost effectiveness: High-precision tool alignment technology often comes with higher costs, requiring a balance between cost and accuracy. Ease of operation: The operation of knife-cutting technology needs to be more convenient to adapt to operators with different skill levels. In the future, research on knife technology will develop in a more intelligent and automated direction to meet the manufacturing industry’s demand for high-precision and high-efficiency processing.
